# Unveiling the local structure of the amorphous metal $\text {Fe}_{(1-x)}\text {Zr}_x$ combining first-principles-based simulations and modelling of EXAFS spectra

**DOI:** 10.1038/s41598-023-32051-3

**Published:** 2023-03-27

**Authors:** Giuseppe Muscas, Robert Johansson, Sebastian George, Martina Ahlberg, Dimitri Arvanitis, Rajeev Ahuja, Ralph H. Scheicher, Petra E. Jönsson

**Affiliations:** 1 Department of Physics & Astronomy, Uppsala University, Box 516, 751 20 Uppsala, Sweden; 2 Department of Physics, University of Cagliari, Cittadella Universitaria di Monserrato, S.P. 8 Km 0.700, 09042 Monserrato, CA Italy; 3 Department of Physics, University of Gothenburg, Fysikgränd 3, 412 96 Gothenburg, Sweden; 4 Indian Institute of Technology Ropar, Rupnagar, Punjab 140001 India; 5 Present Address: Uppsala Clinical Research Center, Uppsala University Hospital, Box 6363, 751 35 Uppsala, Sweden

**Keywords:** Materials science, Condensed-matter physics, Theory and computation

## Abstract

Amorphous alloys exhibit useful properties such as the excellent soft magnetic behaviour of Fe-based metallic glasses. The detailed structure of amorphous $\text {Fe}_{(1-x)}\text {Zr}_x$ with *x* = 0.07, 0.10, and 0.20 is in this work explored through a synergetic combination of atomistic simulations and experimental characterisation. Thin-film samples were investigated using X-ray diffraction and extended X-ray absorption fine structure (EXAFS), while the corresponding atomic structures were simulated using an efficient first-principles-based method called stochastic quenching (SQ). The simulated local atomic arrangements are investigated by constructing the radial- and angular-distribution functions, as well as by Voronoi tesselation. The radial distribution functions are then used to construct a model to fit simultaneously the experimental EXAFS data of multiple samples with different compositions, creating a simple yet accurate description of the atomic structures valid for any composition in the range x = 0.07 to 0.20, using a minimal number of free parameters. This approach significantly improves the accuracy of the fitted parameters and allows us to relate the compositional dependence of the amorphous structures with the magnetic properties. The proposed EXAFS fitting process can be generalised to other amorphous systems, contributing to the understanding of structure-property relationships and the development of amorphous alloys with tailored functional properties.

## Introduction

The magnetic, electrical, and mechanical properties of any material stem from its atomic structure. While a crystalline structure can be completely described by a crystal class and lattice constants, this is not possible for a non-crystalline material, which thus has to be described and characterised in other ways^1,2^. An amorphous material exhibits short-range order and long-range disorder^3–6^. The structure of amorphous materials can be simulated by the stochastic quenching (SQ) method^7–9^, or by the more computationally demanding molecular dynamics simulation method^10^. Simulated amorphous structures have been intensively investigated with focus on the radial distribution function (RDF) and the three-dimensional arrangement of atoms^11–15^. The splitting of the second visible peak in the RDF is a characteristic feature of amorphous metals and it has been explained by considering the next-nearest-neighbour atomic arrangement^1,11–13,15,16^. Recently, it has been attributed to the local quasi-icosahedral ordering in amorphous metals with the first sub-peak of the second peak originating from next-nearest-neighbour atoms with a three-atoms connection of the atomic clusters and the second sub-peak of the second peak originating from next-nearest-neighbour atoms with a one-atom connection of the atomic clusters^12,13^. An accurate and valid atomic structure is a prerequisite for meaningful first-principles modelling of other physical properties. Extended X-ray absorption fine structure (EXAFS) data are sensitive to the short-range order and have been used to qualitatively validate simulated amorphous structures for a few amorphous alloys^9,17,18^. Many details of the atomic structure of amorphous systems are however still unknown.

Metallic glasses are an important class of amorphous materials because of their mechanical and soft-magnetic properties^19^. For magnetic metallic glasses, the Bethe-Slater curve models how the magnetic exchange energy depends on the interatomic distances and thereby provides basic insight into how the magnetic properties depend on the atomic structure^20^. In particular for Fe-based compounds, the magnetic exchange energy depends strongly on the interatomic Fe-Fe distances and the Curie temperature therefore exhibits a strong pressure dependence^21^. Amorphous $\text {Fe}_{(1-x)}\text {Zr}_x$ has been studied extensively as a model system for amorphous iron^22–30^. The magnetic properties have been shown to vary in a nontrivial manner with the chemical composition; for low Zr concentrations ($x \lesssim 0.2$) the Curie temperature increases with increasing Zr content while for higher Zr concentrations it decreases^22,28^. For $\text {Fe}_{(1-x)}\text {Zr}_x$ samples with $x \approx 0.4{-}0.6$, the magnetic properties are strongly affected by the detailed synthesis conditions, and the observed variations in magnetic properties have been attributed to variations in the number of Fe-Fe nearest-neighbours^23,29,30^. For samples prepared by melt-spinning with $x \approx 0.1$, neutron scattering experiments indicate the existence of magnetic inhomogenieties in form of clusters within the ferromagnetic matrix^27^. Still, the amorphous ferromagnetic transition temperature is only slightly affected by structural inhomogenieties such as nanocrystallites^26^. With an optimised sputtering process, it is possible to produce $\text {Fe}_{(1-x)}\text {Zr}_x$ samples without any sign of nanocystallisation^31^.

The aim of this work is to provide a detailed structural analysis of $\text {Fe}_{(1-x)}\text {Zr}_x$ (*x* = 0.07, 0.10, and 0.20). At first, the simulated amorphous structures obtained by the computationally convenient Stochastic Quenching simulation method are analysed. The simulated structures are validated using EXAFS data measured on sputtered samples. To quantify the validation, an advanced fitting process able to pinpoint small differences between the simulated and sputtered amorphous structures is developed. The radial distribution functions obtained for the simulated structures are used to construct a model of the first coordination shell independent of chemical composition to perform the EXAFS fitting. This model can be used to simultaneously fit the experimental EXAFS data of all the samples under investigation, which significantly improves the accuracy and solidity of the EXAFS analysis while using as few fitting parameters as possible.

## Methods

### Experimental

Amorphous $\text {Fe}_{(1-x)}\text {Zr}_x$ samples were grown by DC magnetron sputtering. A $\text {Fe}_{0.93}\text {Zr}_{0.07}$ sample was deposited from a compound target and the details about the sample preparation are given in Ref.^32^. $\text {Fe}_{0.90}\text {Zr}_{0.10}$ and $\text {Fe}_{0.80}\text {Zr}_{0.20}$ samples were deposited via co-sputtering from individual Fe and Zr targets at room temperature on 3$''$ Si (001) substrates. The substrates were annealed at $650\,^\circ \text {C}$ for 30 min prior to deposition in order to clean the surface. The base pressure of the chamber was below $8\times 10^{-10}$ Torr, while the growth was conducted with an Ar gas (99.9999 % pure) pressure of $2\times 10^{-3}$ Torr and substrate rotation. To facilitate the growth of completely amorphous $\text {Fe}_{(1-x)}\text {Zr}_x$, a buffer layer of amorphous $\text {Al}_{0.70}\text {Zr}_{0.30}$ (5 nm thick) was used, as well as a capping layer of $\text {Al}_{0.70}\text {Zr}_{0.30}$ (4 nm thick) to protect the samples against oxidation^31^. The compositions were confirmed to be within ±0.5 at.% of the nominal values by Rutherford backscattering spectrometry (RBS). The analysis of the RBS spectra was performed using the SIMNRA simulation software^33,34^.

X-ray reflectivity (XRR) measurements were collected in the 2$\theta$ range 0.2–$8.0^\circ$ with a Bruker D8 Da Vinci diffractometer. With the same instrument, grazing-incidence X-ray diffraction (GIXRD) patterns were collected in the 2$\theta$ range 10–$80^\circ$, with an incident angle of $1^\circ$. The measured XRR patterns were fitted using the GenX simulation software^35^, in order to determine the thicknesses of the individual layers as well as their number densities. The results are presented in Table 1 together with values of the number densities reported in the literature^36,37^. The GIXRD data (Fig. S1 in the supplementary information) confirm the x-ray amorphous character of all the samples.

**Table 1:** The thicknesses and number densities obtained by fitting the XRR patterns using the GenX software^35^.

Composition	Thickness (Å)	Number density, $\rho _N$ (Å$^{-3}$)	
	XRR fit	XRR fit	Refs.^36,37^
$\text {Fe}_{0.93}\text {Zr}_{0.07}$	400(5)	0.080(2)	0.080
$\text {Fe}_{0.90}\text {Zr}_{0.10}$	999(5)	0.078(2)	0.078
$\text {Fe}_{0.80}\text {Zr}_{0.20}$	758(5)	0.070(3)	0.073

The number density values reported in the literature^36,37^ are also included.

Extended X-ray absorption fine structure (EXAFS) measurements were carried out at 80 and 300 K, using X-ray synchrotron radiation at beamline I811 at MAX-lab (Lund, Sweden). The data was collected in fluorescence mode over the K edge of Fe using a Si(111) double-crystal monochromator and an energy-dispersive solid-state detector (Si Nano Technology Vortex with a resolution $\Delta E/E$ of the order $10^{-4}$). The data was obtained by averaging eight 40-minute scans per sample to improve the signal-to-noise ratio. The normalised EXAFS functions $\chi (k)$ were extracted from the raw data following standard procedures using the ATHENA software^38^. Hanning windows are used for the Fourier transform with $k \sim 2.4{-}10.7$ Å$^{-1}$ for the amorphous samples and $2.4{-}12.5$ Å$^{-1}$ for the Fe film. Unfortunately, it was not possible to measure over the K-edge of Zr with enough accuracy due to experimental limitations. The measurements at 80 and 300 K did not significantly differ, except for the expected reduction of the thermal disorder and hence a slightly better signal-to-noise ratio in the low-temperature data. Therefore, the investigation was focused on the 80 K data.

### Simulated amorphous structures

The simulated amorphous structures of $\text {Fe}_{(1-x)}\text {Zr}_x$ ($x=0.07$, 0.10, and 0.20) were created using the SQ method^7–9^. The SQ method initiates from a random starting configuration of atoms and relaxes the positions of the atoms to the nearest local minimum in the potential-energy landscape. By repeatedly carrying out this process for a number of different random starting positions, we create samples of the amorphous structure, which are then subsequently used to obtain averages of the investigated properties. Due to the nature of the potential energy landscape, a structure which has been relaxed to the nearest local minimum from a random starting configuration is a good representation of an amorphous structure for the given composition of elements. The corresponding experimental sample would be one quenched from a melt with a very rapid cooling rate, or in the case of sputtering, be one with the kinetic energy of the adatoms perfectly tuned both to avoid any kind of clustering and to avoid porous growth.

The only experimental input to the simulations was the values of the number densities that were chosen according to the literature values reported in Table 1^36,37^. The atoms are initially stochastically distributed into a cubic supercell with the only constraint being that the nearest-neighbour distance must be larger than a chosen minimum distance. The relaxation of the atomic structures to nearby energy minima was performed using the Vienna *Ab initio* Simulation Package (VASP)^10,39–41^. The interactions between the electrons and the nuclei were obtained using the projector-augmented-wave method^42,43^. The generalised gradient approximation (GGA) in the parametrisation of Perdew-Burke-Ernzerhof (PBE)^44,45^ approach was employed to approximate the exchange and correlation terms in the density functional theory (DFT) method^46,47^. A conjugate gradient algorithm was used to relax the atomic nuclei positions to a local minimum in the total energy landscape. Due to the large size of the supercell, it was considered appropriate to sample the $\Gamma$-point only in the Brillouin zone. A plane-wave energy cutoff of 400 eV was used. Initially, the minimum nearest neighbour distance was 1.4 Å. Comparisons with structures generated from the relaxation of initial stochastic configurations with a constraint of 0.5 Å for the nearest neighbour minimum distance yielded no notable difference in structural properties while the constraint of 1.4 Å offered a substantial improvement in computational time. The amorphous $\text {Fe}_{(1-x)}\text {Zr}_x$ structures were for each composition modelled by the atomic relaxation of at least 50 random starting configurations of supercells, each containing in total 200 atoms. The details about the supercells including their size are reported in Table S1 in the supplementary information.

Voronoi tessellation was performed using the Voro++ software package^48^. The difference in size between the Fe and Zr atoms is taken into account when creating the Voronoi polyhedra. The Voronoi indices are presented in the form $\langle n_3,n_4,n_5,n_6\rangle$ where $n_i$ denotes the number of surfaces with *i* number of edges. $\sum \limits _i n_i$ gives the Voronoi coordination number. Voronoi polyhedra surfaces with an area that comprises less than 1$\%$ of the total area of the polyhedra are disregarded.

**Figure 1:**
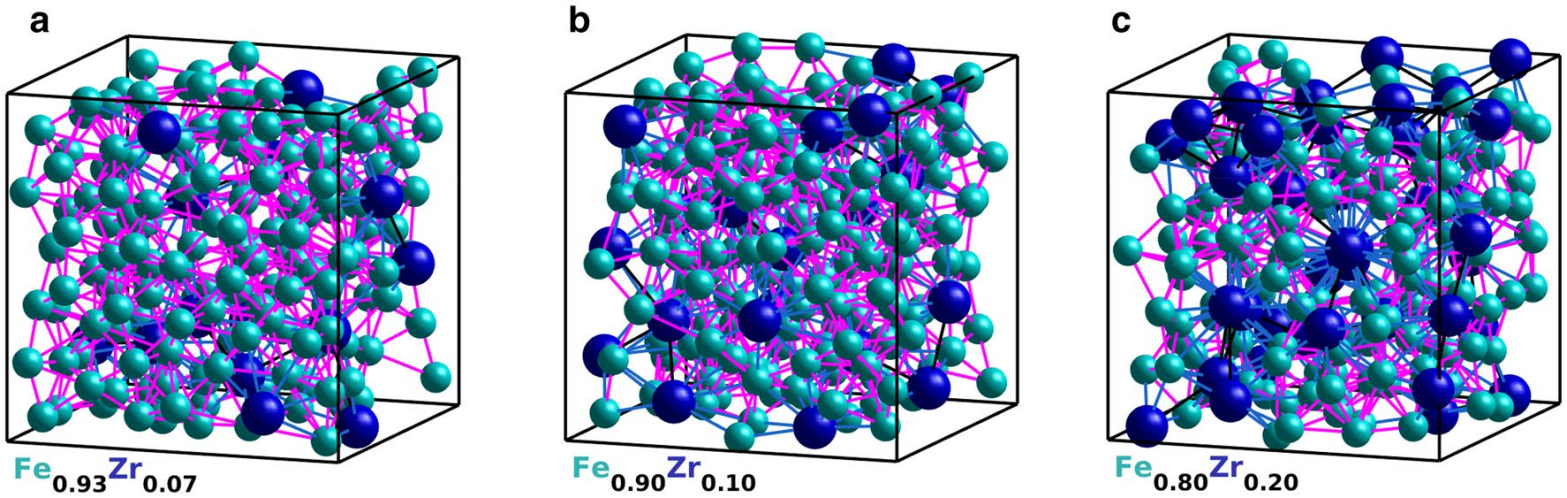
Illustration of the atomic arrangement in one of the simulated supercells for $\text {Fe}_{0.93}\text {Zr}_{0.07}$, $\text {Fe}_{0.90}\text {Zr}_{0.10}$, and $\text {Fe}_{0.80}\text {Zr}_{0.20}$ from left to right. The larger dark blue spheres represent Zr atoms and the smaller cyan spheres represent Fe atoms. Nearest neighbour bounds are indicated by lines (magenta for Fe-Fe, blue for Fe-Zr, and black for Zr-Zr). The simulations used periodic boundary conditions.

## Results

### The simulated amorphous structures

**Figure 2:**
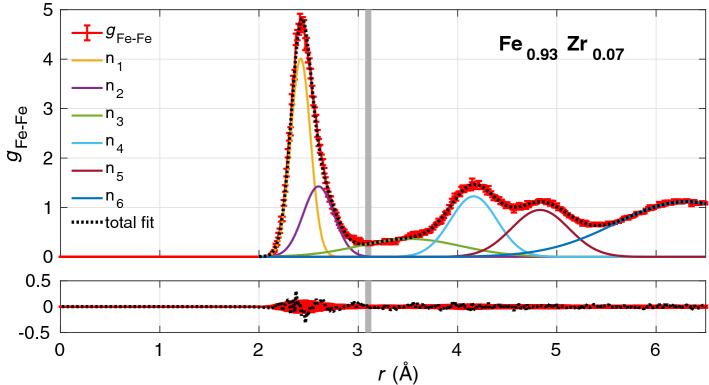
Partial radial distribution function for Fe-Fe pairs in $\text {Fe}_{0.93}\text {Zr}_{0.07}$. The lower panel shows the residual between the sum of the six Gaussians and $g_{\mathrm{Fe-Fe}}(r)$ obtained from the simulated supercells. The gray vertical line indicates the limit of the first shell. In the lower panel, the difference between the total fit and $g_{\mathrm{Fe-Fe}}$ are shown as black points while the standard deviation of the simulated partial radial distribution is indicated in red.

**Figure 3:**
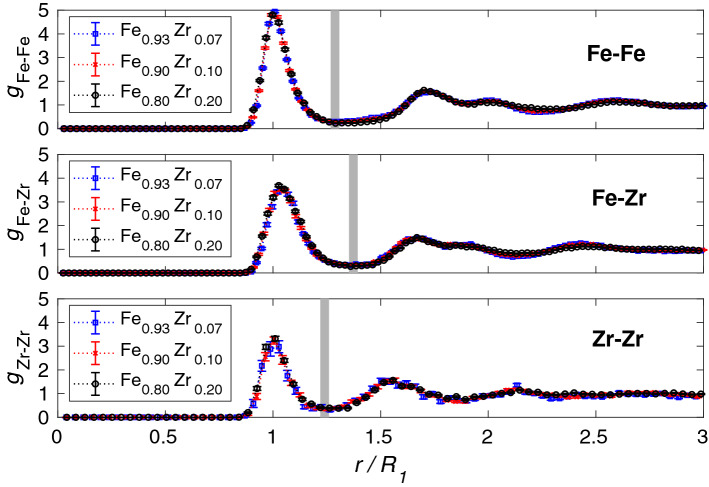
Partial radial distribution functions (RDFs) obtained from the simulated atomic positions for Fe-Fe, Fe-Zr, and Zr-Zr pairs versus $r/R_1$. The $R_1$ values are reported in Table 2. The thick gray vertical lines indicate the limit of the first coordination shells.

Representative examples of the simulated amorphous structures, generated with periodic boundary conditions, are shown in Fig. 1 for one of the supercells of each composition. The partial radial distribution functions (RDF) were calculated for Fe-Fe, Fe-Zr, and Zr-Zr pairs from the atomic positions in each supercell and then averaged using all the simulated supercells. Each partial RDF, *g*(*r*), is normalised so that $g(r \rightarrow \infty ) =1$, in order to be able to directly compare the partial RDFs from simulations with different compositions. Next, all the partial RDFs for the different compositions were parameterised by fitting a sum of *N* Gaussians

1 $$\begin{aligned} g(r)=\sum _i^N a_i n_i(r;R_i,\sigma _i), \; \; \; n_i(r;R_i,\sigma _i)= \frac{1}{\sqrt{2 \pi \sigma _i^2}} \exp {\left( -\frac{(r-R_i)^2}{2 \sigma _i^2}\right) }, \end{aligned}$$

where $a_{i}$ is the amplitude, $R_{i}$ the peak position, and $\sigma _{i}$ the standard deviation of the *i*th Gaussian $n_i$. A fit of $g_{\mathrm{Fe-Fe}}(r)$ for $\text {Fe}_{0.93}\text {Zr}_{0.07}$ is shown in Fig. 2. The fit is within the statistical uncertainty of the partial RDF for almost all values of *r* as can be seen in the lower panel of the figure. All partial RDFs for the three compositions were fitted and the peak positions of the first Gaussian, $R_{1}$, are reported in Table 2. We note that all values of $R_1$ decrease with increasing Zr content, but, the relative positions of the other peaks are independent of composition for the simulated structures. To illustrate this, all partial RDFs are plotted versus $r/R_1$ in Fig. 3. At large *r* values, the RDFs flatten out due to the long-range disorder of the amorphous structures. A clear splitting of the second peak is seen for $g_{\mathrm{Fe-Fe}}$ and $g_{\mathrm{Fe-Zr}}$. A summary of the relative peak positions ($R_{i}/R_1$) obtained by fitting the Gaussians is reported in Table 3. Note that the $R_4$ and $R_5$ peaks are the first and second sub-peaks of the second visible peak of the RDF.

**Table 2:** Peak positions of the first Gaussian, $R_1$, obtained fitting Eq. (1) to the partial Fe-Fe, Fe-Zr, and Zr-Zr radial distribution functions for the simulated amorphous structures of $\text {Fe}_{0.93}\text {Zr}_{0.07}$, $\text {Fe}_{0.90}\text {Zr}_{0.10}$, and $\text {Fe}_{0.80}\text {Zr}_{0.20}$.

	$R_1^{\mathrm{Fe-Fe}}$ (Å)	$R_1^{\mathrm{Fe-Zr}}$ (Å)	$R_1^{\mathrm{Zr-Zr}}$ (Å)
$\text {Fe}_{0.93}\text {Zr}_{0.07}$	2.419(4)	2.79(3)	3.43(3)
$\text {Fe}_{0.90}\text {Zr}_{0.10}$	2.411(4)	2.78(2)	3.40(2)
$\text {Fe}_{0.80}\text {Zr}_{0.20}$	2.389(4)	2.73(2)	3.33(2)

**Table 3:** Relative peak positions of the *i*th Gaussian obtained by fitting Eq. (1) to the partial Fe-Fe, Fe-Zr, and Zr-Zr radial distribution functions (RDF) for the simulated amorphous structures of $\text {Fe}_{0.93}\text {Zr}_{0.07}$, $\text {Fe}_{0.90}\text {Zr}_{0.10}$, and $\text {Fe}_{0.80}\text {Zr}_{0.20}$. For Fe-Fe and Fe-Zr, the $R_4$ and $R_5$ values are the peak positions of the first and second sub-peaks of the second visible peak of the partial RDFs. Fewer Gaussians are used to fit the Zr-Zr partial RDF and values for $R_{3}$ and $R_{5}$ are therefore missing so that the $R_4$ value is the peak position of the second visible peak in the Zr-Zr partial RDF.

	$R_{2}/R_{1}$	$R_{3}/R_{1}$	$R_{4}/R_{1}$	$R_{5}/R_{1}$
Fe-Fe	1.08(1)	1.46(4)	1.72(2)	2.00(2)
Fe-Zr	1.07(3)	1.2(1)	1.64(2)	1.82(2)
Zr-Zr	1.10(8)	–	1.56(4)	–

**Figure 4:**
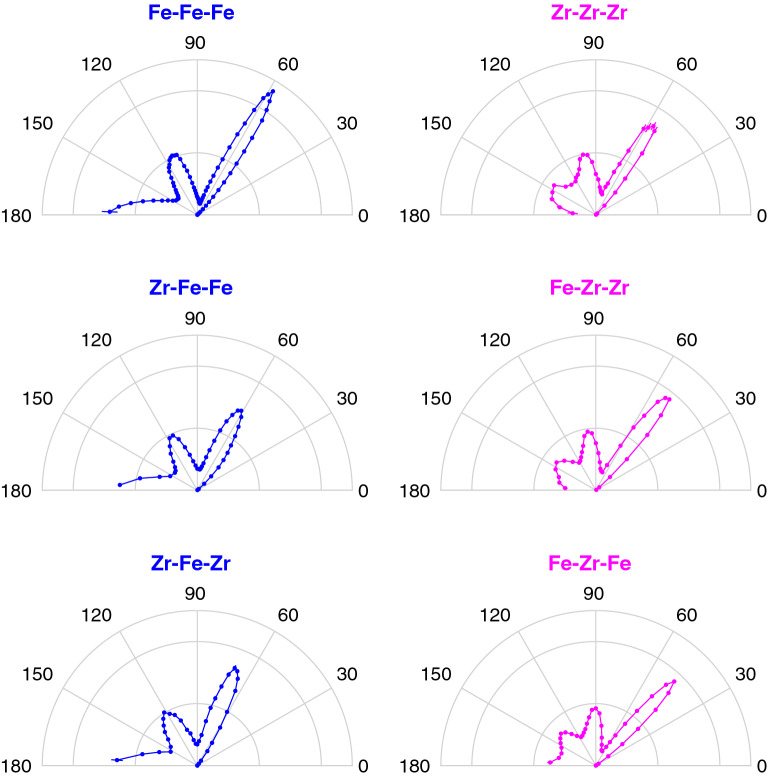
Polar plots of the partial angular distribution functions obtained from the simulated atomic positions for atoms belonging to the first shell around the central atom. These plots look quite similar for all compositions, but are only shown here for $\text {Fe}_{0.80}\text {Zr}_{0.20}$ due to the better statistics for Zr in that case. Error bars are plotted, but are generally smaller than the size of the data points. The position of the cut-off for the first shell is indicated as a gray line for each partial radial distribution function in Fig. 3.

To further investigate the geometrical arrangement of atoms, the partial angular distribution functions (ADFs) were calculated from all *A*-*B*-*C* atomic trios, where *A* and *C* are both within the nearest-neighbour shell around the central atom *B*. If each angle $\theta$, defined as the angle between the *B*-*A* and *B*-*C* vectors, is equally probable due to a random arrangement of atoms, the probability distribution will exhibit a $\sin ( \theta )$ dependence. Therefore, the probability at each angle $\theta$ is normalised by $\sin ( \theta )^{-1}$ in order to give equal weight to each angle. The partial ADFs are shown in Fig. 4. With Fe as the central atom, the first peak is close to $60^\circ$ for the Fe-Fe-Fe trios, indicating an equilateral triangular arrangement if only atoms of the same size are involved. If both Fe and Zr atoms are included, the angles deviate from $60^\circ$ in accordance with larger Zr atoms and smaller Fe atoms. The minimum at $90^\circ$ indicates that a cubic arrangement of atoms around Fe is unfavourable, while the additional peak in the *A*-Fe-*C* ADFs close to $117^\circ$ is consistent with an icosahedral ordering of the atoms. It is only Fe-atoms surrounding Zr atoms that according to the Fe-Zr-Fe ADF have a tendency for cubic arrangements with peaks close to $45^\circ$, $90^\circ$, $135^\circ$ and $180^\circ$. The first (lowest angle) peak in the *A*-*B*-*C* ADF contributes to the nearest-neighbor peak in $g_{C-A}$. For the *A*-Fe-Fe ADFs, it is the two atoms with an angle close to $117^\circ$ to the central atom that give rise to the $R_4$-peak in $g_{\textrm{Fe}-A}$ and those with an angle close to $180^\circ$ that give rise to the $R_5$-peak. The Zr-Zr-Zr ADF exhibits a minimum at $180^\circ$, indicating that Zr-atoms do not tend to form linear arrangements and explains the absence of $R_5$-peak in $g_{\mathrm{Zr-Zr}}$. A Voronoi analysis was performed to provide more information about the three-dimensional arrangement of the atoms. It revealed, in accordance with other metallic glasses^6^, a quasi-icosahedral ordering with typical Voronoi indices $\langle 0,1,10,2\rangle$, $\langle 0,2,8,2\rangle$, $\langle 0,3,6,4\rangle$, and $\langle 0,0,12,0\rangle$ for Fe atoms and $\langle 0,1,10,7\rangle$ for Zr atoms.

### EXAFS analysis

**Figure 5:**
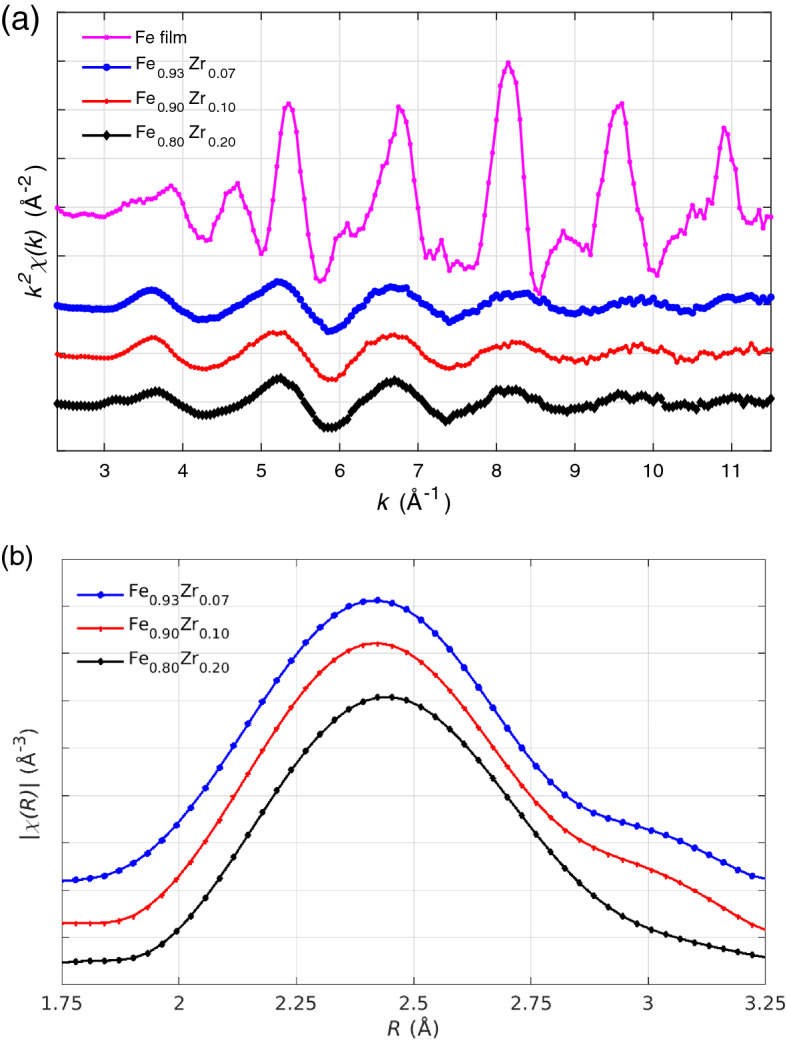
(**a**) EXAFS signal $k^2 \chi$ measured at 80 K for $\text {Fe}_{0.93}\text {Zr}_{0.07}$, $\text {Fe}_{0.90}\text {Zr}_{0.10}$, $\text {Fe}_{0.80}\text {Zr}_{0.20}$, and the reference crystalline BCC-Fe film. (**b**) Its Fourier transform plotted with phase-correction for the three amorphous samples. All data are shifted vertically for clarity with the zero levels indicated by the grid.

The $k^2$-weighted Fe K-edge EXAFS signals, measured at 80 K, are shown in Fig. 5a for the amorphous $\text {Fe}_{(1-x)}\text {Zr}_x$ samples as well as for a crystalline BCC-Fe film as a reference. The magnitude of the Fourier transform of the $k^2 \chi (k)$ data are shown in Fig. 5b for the amorphous samples. The amplitude and accuracy of the $\chi (R)$ data beyond the first shell is very low and can not be included in any analysis.

In a first attempt to determine if the simulated atomic structures are compatible with the experimental EXAFS data, the average $\chi (q)$ function was calculated, using the FEFF8.50-Lite code^49,50^, from the exact atomic positions in all supercells. Only atoms within the nearest-neighbour shell around each Fe atom were included in the calculation and periodic boundary conditions were applied. The amplitude $S_0^2$ and the energy shift $\Delta E_0$ were fitted to the experimental data simultaneously for *k*-weights of 1, 2, and 3. The simulated and experimental $k^2\chi (q)$ data are shown in Fig. S2 in the Supplementary Information. They are quite similar for $\text {Fe}_{0.93}\text {Zr}_{0.07}$ and $\text {Fe}_{0.90}\text {Zr}_{0.10}$, but clearly deviate for $\text {Fe}_{0.80}\text {Zr}_{0.20}$. We repeated the SQ simulation with a slightly lower number density for $\text {Fe}_{0.80}\text {Zr}_{0.20}$ (close to that obtained in the fit of the XRR data reported in Table 1). However, the simulated EXAFS signal was only slightly changed and the fit to the experimental data was not improved. Hence, an incorrect density in the simulation is not the reason for the discrepancy between the simulated and experimental EXAFS signal for the $\text {Fe}_{0.80}\text {Zr}_{0.20}$ sample.

**Figure 6:**
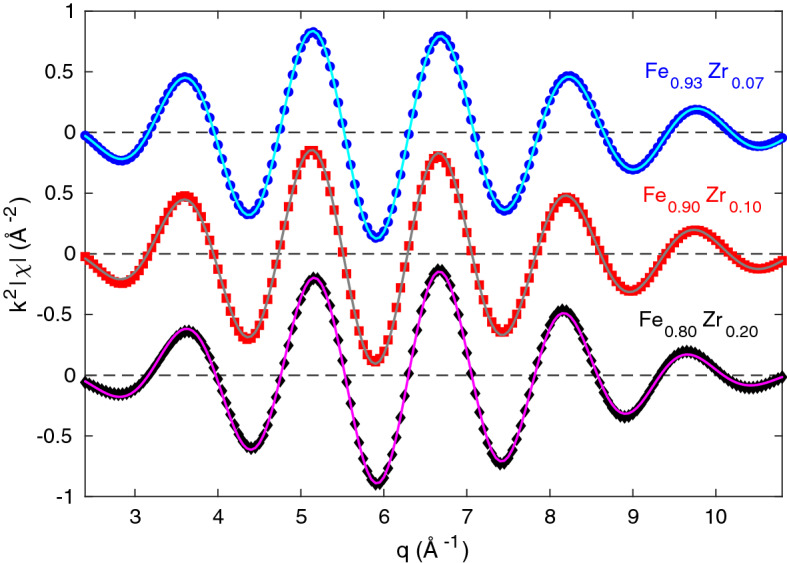
$k^2$-weighted real part of $\chi (q)$ for experimental data (points) measured at 80 K and fits according to the four path model described in the text (lines). Both the experimental data and the fits are limited to the first shell. The back-Fourier analysis is made for $R \sim 1.5{-}2.7$ Å using a Hanning window for the experimental data (corresponding to non-phase corrected distances). The data are shifted for clarity with the dashed lines indicating each zero level.

In order to understand the differences between the simulated amorphous structures and those of the experimental samples, we have created a model to fit to the experimental EXAFS data for all samples. This model is based on the simulated partial Fe-Fe and Fe-Zr RDFs and uses the observation in Fig. 3 that the scaled partial RDFs are the same for all three compositions. Parameters from the Gaussian fits with $i=1$ and 2 in Eq. (2) for $g_{\mathrm{Fe-Fe}}$ and $g_{\mathrm{Fe-Zr}}$ were used to construct the model consisting of 4 paths in total. The partial coordination number of each path is given by

2 $$\begin{aligned} N_i^{A-B}=4 \pi c_B \rho _N a_i\int _0^\infty \frac{ r^2}{\sqrt{2 \pi \sigma _i^2} } \exp {\left( -\frac{(r-R_i)^2}{2 \sigma _i^2}\right) } dr = 4 \pi \rho _B a_i ( \sigma _i^2 + R_i^2). \end{aligned}$$

where $\rho _B=c_B \rho _N$, $c_B$ is the atomic content of element *B*, and $\rho _N$ is the number density that is given in Table S1 (Supplementary Information) for the three compositions. $a_{i}$ is the amplitude, $R_{i}$ the peak position, and $\sigma _{i}$ the standard deviation of the *i*th Gaussian ($i=1$ or 2) according to Eq. (1). In order to further reduce the number of fitting parameters, the scaled Debye-Waller factors are assumed to be the same for the Fe-Fe and Fe-Zr paths of the same order, since the fitted values indicate that they are very similar. In the model, the scaled Fe-Fe and Fe-Zr coordination numbers are fixed to the values obtained by the Gaussian fits The EXAFS $\chi (q)$ data belonging to the first shell were fitted simultaneously for all three samples using the ARTEMIS software package with a simultaneous k-weight of 1, 2, and 3, with some fitting variables in common for the three compositions. The values of all parameters that were fitted separately for the three $\text {Fe}_{(1-x)}\text {Zr}_x$ compositions are reported in Table 4 together with those obtained fitting a BCC structure to the Fe reference film. The values of the scaled parameters in the four EXAFS paths that are common for all samples are reported in Table 5 together with the corresponding values obtained for the two first Gaussians, when fitting $g_{\mathrm{Fe-Fe}}$ and $g_{\mathrm{Fe-Zr}}$ of the simulated structures to Eq. (1). The $k^2$-weighted $|\chi (q)|$ data and the fits are shown in Fig. 6 for the three FeZr samples.

The main part of the EXAFS signal comes from Fe-Fe contributions and only a minor part from Fe-Zr contributions. Still, the Fe-Zr paths are needed in order to obtain good fits, though the parameter values from the Fe-Zr paths have higher uncertainty than those from the Fe-Fe paths. For the $\text {Fe}_{0.80}\text {Zr}_{0.20}$ sample, with the largest contribution from the Fe-Zr paths, $R_1^{\mathrm{Fe-Zr}}$ was included as a fitting parameter and found within the uncertainties to be the same as the simulated value reported in Table 2. For the $\text {Fe}_{0.93}\text {Zr}_{0.07}$ and $\text {Fe}_{0.90}\text {Zr}_{0.10}$ samples, with lower contributions from the Fe-Zr paths, the $R_1^{\mathrm{Fe-Zr}}$ distances were taken from the Gaussian fits. Allowing the radial distances of the second path $R_2/R_1$ to vary, did not really improve the quality of the fit for either Fe-Fe or for Fe-Zr. The values were therefore taken from the Gaussian fits of the simulated partial RDFs. For the $R_1^{\mathrm{Fe-Fe}}$ values, the fitted and simulated values are very close for $\text {Fe}_{0.93}\text {Zr}_{0.07}$. However, as the Zr concentration increases, the simulated $R_1^{\mathrm{Fe-Fe}}$ distance decreases, while the EXAFS fitted $R_1^{\mathrm{Fe-Fe}}$ distance increases (see Fig. S3 in the Supplementary Information). The trend of increasing Fe-Fe distance with increasing Zr content agrees with the magnetic properties; The Curie temperature increases with increasing Zr content for the $\text {Fe}_{(1-x)}\text {Zr}_x$ samples (see Table S2 in the Supplementary Information) and a higher Curie temperature is related to a larger Fe-Fe distance according to the Bethe-Slater curve^20^.

To summarise, the Fe-Fe distances are the biggest source of discrepancy between the simulated and real amorphous structures, a difference that increases with increasing Zr content. Due to the low Zr content, we cannot draw any conclusions about the Fe-Zr distances. We only note that the uncertainties are of the same order as the discrepancies in Fe-Fe distances. The two-path approach is a simple way of obtaining an asymmetric distribution of bond-lengths. Allowing $N_1/c_B$ to vary while keeping both the $R_2/R_1$ ratios as well as the total coordination numbers fixed allow to modify this asymmetry without making the fitting procedure unstable. The coordination numbers scaled by the atomic content are in principle allowed to vary indirectly for each samples since the amplitude factors $S_0^2$ are fitted individually. However, the fitted $S_0^2$ values are within the uncertainties the same for all samples.

**Table 4:** The parameter values obtained from the EXAFS fits that are different for each sample.

	$R_1^{\mathrm{Fe-Fe}}$ (Å)	$R_1^{\mathrm{Fe-Zr}}$ (Å)	$S_0^2$	$\Delta E_0 (eV)$
$\text {Fe}_{0.93}\text {Zr}_{0.07}$	2.432(8)	*2.79*	0.54(7)	4(2)
$\text {Fe}_{0.90}\text {Zr}_{0.10}$	2.440(7)	*2.78*	0.58(7)	4(2)
$\text {Fe}_{0.80}\text {Zr}_{0.20}$	2.442(8)	2.75(3)	0.60(7)	5(1)
Fe (BCC)	2.481(5)	–	0.7(1)	6(1)

The $R_1^{\mathrm{Fe-Zr}}$ values for $\text {Fe}_{0.93}\text {Zr}_{0.07}$ and $\text {Fe}_{0.90}\text {Zr}_{0.10}$ were fixed to those reported in Table 2 (written here in italic). The Fe-film data are fitted with a BCC structure in the range $R = 1.8{-}4.9$ Å (non-phase corrected) using a Hanning window.

**Table 5:** Scaled parameters used in the EXAFS fits that are common for all compositions.

	From *g*(*r*)		EXAFS fit	
	Fe-Fe	Fe-Zr	Fe-Fe	Fe-Zr
$R_2/R_1$	1.08(2)	1.07(3)	*1.08*	*1.07*
$\sigma _1^2/R_1^2$	0.0018(3)	0.0017(6)	0.0016(2)	
$\sigma _2^2/R_1^2$	0.0045(20)	0.0035(21)	0.0014(7)	
$N_1/c_B$	6.4(8)	4.7(2.9)	8(1)	11(2)
$(N_1+N_2)/c_B$	10.4(0.8)	13.3(1.0)	*10.4*	*13.3*

The actual parameters that are used in the EXAFS paths ($R_2$, $\sigma ^2_1$. $\sigma ^2_2$, $N_1$, and $N_2$) are different for all concentrations since they are scaled by either $R_1^{A-B}$ or the *B* atom content ($c_B$). The errorbars are estimated from the compositional variations. The parameter values are obtained from the fitting to the experimental EXAFS data except those written in italic that were fixed to the value obtained in the Gaussian fitting of the simulated partial radial distribution functions, *g*(*r*).

### Coordination numbers

The Fe-Fe and Fe-Zr partial coordination numbers in the EXAFS fits are obtained by summing up those of the two paths and correcting for $S_0^2$. These values are reported in Table 6 and compared to the coordination numbers obtained from the simulated structures using three different methods: (i) summing up those of the Gaussians with $i=1$ and 2 obtained according to Eq. (2), (ii) from the partial RDF as

3 $$\begin{aligned} N^{A-B}=4\pi \rho _B \int _0^{r_{\textrm{min}}} r^2 g(r) dr, \end{aligned}$$

where the upper limit of the integral is the radial distance $r_{\textrm{min}}$ corresponding to the minimum in *g*(*r*), and (iii) from a Voronoi analysis. Somewhat different coordination numbers are obtained with the different methods. It is, however, not surprising that lower coordination numbers are obtained from the Gaussians with $i=1$ and 2 (Eq. 2) compared to integrating the partial RDFs up to $r_{\textrm{min}}$ (Eq. 3) if one looks at Fig. 2, where the limit of the first shell ($r_{\textrm{min}}/R_1$) is indicated with a gray line. Due to the difference in obtained values depending on the method, the large uncertainties, and the known difficulty to obtain accurate coordination number of amorphous compounds from EXAFS data, we cannot state a significant discrepancy between the simulated and experimental coordination numbers.

**Table 6:** Nearest-neighbour coordination numbers obtained from the fitting of the EXAFS data and from the simulated structures.

		EXAFS fit	Equation (2)	Equation (3)	Voronoi
$N^{\mathrm{Fe-Fe}}$	$\text {Fe}_{0.93}\text {Zr}_{0.07}$	7(2)	9.6(1)	10.5(3)	11.9(4)
	$\text {Fe}_{0.90}\text {Zr}_{0.10}$	8(2)	9.3(3)	10.0(2)	11.2(4)
	$\text {Fe}_{0.80}\text {Zr}_{0.20}$	7(2)	7.7(2)	8.3(2)	9.1(4)
$N^{\mathrm{Fe-Zr}}$	$\text {Fe}_{0.93}\text {Zr}_{0.07}$	0.7(2)	0.9(2)	1.2(1)	1.3(1)
	$\text {Fe}_{0.90}\text {Zr}_{0.10}$	1.1(3)	1.3(1)	1.8(1)	1.8(1)
	$\text {Fe}_{0.80}\text {Zr}_{0.20}$	2.2(5)	2.5(3)	3.3(1)	3.4(1)
$N^{\mathrm{Zr-Zr}}$	$\text {Fe}_{0.93}\text {Zr}_{0.07}$			1.5(1)	1.6(2)
	$\text {Fe}_{0.90}\text {Zr}_{0.10}$			2.1(2)	2.2(2)
	$\text {Fe}_{0.80}\text {Zr}_{0.20}$			4.1(3)	4.3(3)

The coordination numbers of the simulated structures are calculated in three different ways: by summing $N^{A-B}_1$ and $N^{A-B}_2$ calculated from Eq. (2), from Eq. (3), and from a Voronoi analysis. Details about the distribution of Voronoi coordination numbers are presented in Fig. S4 in the Supplementary Information.

## Discussion

While many investigations of amorphous structures are based on independent theoretical or experimental observations, this work provides a detailed structural analysis of Fe-based amorphous alloys combining the theoretical information obtained by the computationally convenient SQ simulation method and the information extracted with an advanced fitting process from experimental EXAFS data. The simulation-enhanced fitting model surpasses the limitation of conventional EXAFS fitting and allows us to predict the compositional dependence of the magnetic properties for the investigated samples. At the same time, the results provide further confirmation of the validity of the SQ approach. Nonetheless, while we have found overall very good agreement between simulation and experiment, there are some discrepancies, accentuated by increasing the Zr content. One potential explanation is that in the sputtered samples, the Fe atoms might tend to be inhomogeneously distributed, as previously reported for $\text {Fe}_{(1-x)}\text {Zr}_x$ samples with high Zr content^23,29,30^. We note that this inhomogeniety is not the same as the formation of crystallites. It is rather a small deviation from the ”most random” or most homogeneous amorphous structure due to the kinetics of the sputtered atoms and differences in glass forming ability with Zr-content. In the simulations, due to the nature of the stochastic quenching method, we rather expect a quite homogeneous amorphous structure independent of Zr-content. A second potential explanation, that we cannot exclude, is that the employed density functional theory implementation possesses some inherent quality differences between the pseudopotentials for Fe and Zr, causing the electron density distribution around Zr atoms to be slightly less accurate than that for Fe. These two hypotheses deserve further attention and indicate areas of improvement for simulations of real amorphous systems. However, this does not undermine the general validity of the simulation-enhanced fitting approach here proposed, and it does not prevent its application to other amorphous alloys, in particular in the case of low-concentration of the glassy former. This is also the most technologically relevant case at least for Fe-based amorphous soft-magnetic alloys.

## Conclusions

To shed light on the complex structure of amorphous metals, a combination of theoretical modelling and experimental characterisation was used to study amorphous $\text {Fe}_{(1-x)}\text {Zr}_x$ with $x = 0.07$, 0.10, and 0.20. The partial radial distribution functions of the simulated atomic structures were used to create a model to simultaneously fit the experimental EXAFS data of all three samples to have a simple but accurate description of the atomic structures valid for any composition in the range $x=0.07{-}0.20$ using a minimal number of free parameters. This approach allowed to extend the investigation beyond the individual limits of the theoretical model and the experimental data, revealing details of the structure not easily accessible. The EXAFS fitting revealed that the trend of the simulations of a slightly decreasing $R_1^{\mathrm{Fe-Fe}}$ with increasing Zr content is incorrect and that in fact, $R_1^{\mathrm{Fe-Fe}}$ increases slightly with increasing Zr content. The magnetic properties are highly sensitive to the nearest-neighbour Fe-Fe distances and the increase of Curie temperature with increasing Zr content is consistent with an increase in $R_1^{\mathrm{Fe-Fe}}$ according to the Bethe-Slater curve^20^.

A detailed amorphous structural description with accurate inter-atomic distances is a prerequisite for first-principles predictions of electronic and magnetic properties. The process outlined in this work can be generalised to other amorphous systems and is well suited also for multi-component glasses since the knowledge about the simulated structures is used to reduce the complexity and the number of parameters included in the fitting of the experimental EXAFS data. Further combined experimental and atomic simulation studies could therefore pave the way for detailed investigations of the relationship between amorphous structures and physical properties. Such knowledge about the structure-properties relationship would be a valuable guide in the development of new amorphous materials with tailored functional properties.

## Supplementary Information

Supplementary Information.

## Data Availability

Request for any data should be addressed to the corresponding author (P.E.J.).
