# Supplementary material for: Unveiling the local structure of the amorphous metal Fe(1-x)Zrx combining first-principles-based simulations and modelling of EXAFS spectra
Source: Sci Rep. 2023 Mar 27;13:4983. doi: 10.1038/s41598-023-32051-3 (PMC10042867; doi:10.1038/s41598-023-32051-3)
Supplement: Supplementary file 1 — Supplementary Information. [file 41598_2023_32051_MOESM1_ESM.pdf]

Supplementary Information

Unveiling the local structure of the amorphous metal $Fe_{(1-x)}Zr_x$ combining first-principles-based simulations and modelling of EXAFS spectra

Giuseppe Muscas,[1,2] Robert Johansson,[1,3] Sebastian George,[1] Martina Ahlberg,[1,4] Dimitri Arvanitis,[1] Rajeev Ahuja,[1,5] Ralph H. Scheicher,[1] and Petra E. Jönsson[1,6]

[1]*Department of Physics & Astronomy, Uppsala University, Box 516, SE-751 20 Uppsala, Sweden*

[2]*Present address: Department of Physics, University of Cagliari, Cittadella Universitaria di Monserrato, S.P. 8 Km 0.700, I-09042 Monserrato (CA), Italy*

[3]*Present address: Uppsala Clinical Research Center, Uppsala University Hospital, Box 6363, SE-751 35 Uppsala, Sweden*

[4]*Present address: Department of Physics, University of Gothenburg, Fysikgränd 3, SE-412 96 Gothenburg, Sweden*

[5]*Indian Institute of Technology Ropar, Rupnagar,Punjab - 140001,India*

[6]*Corresponding author: petra.jonsson@physics.uu.se*

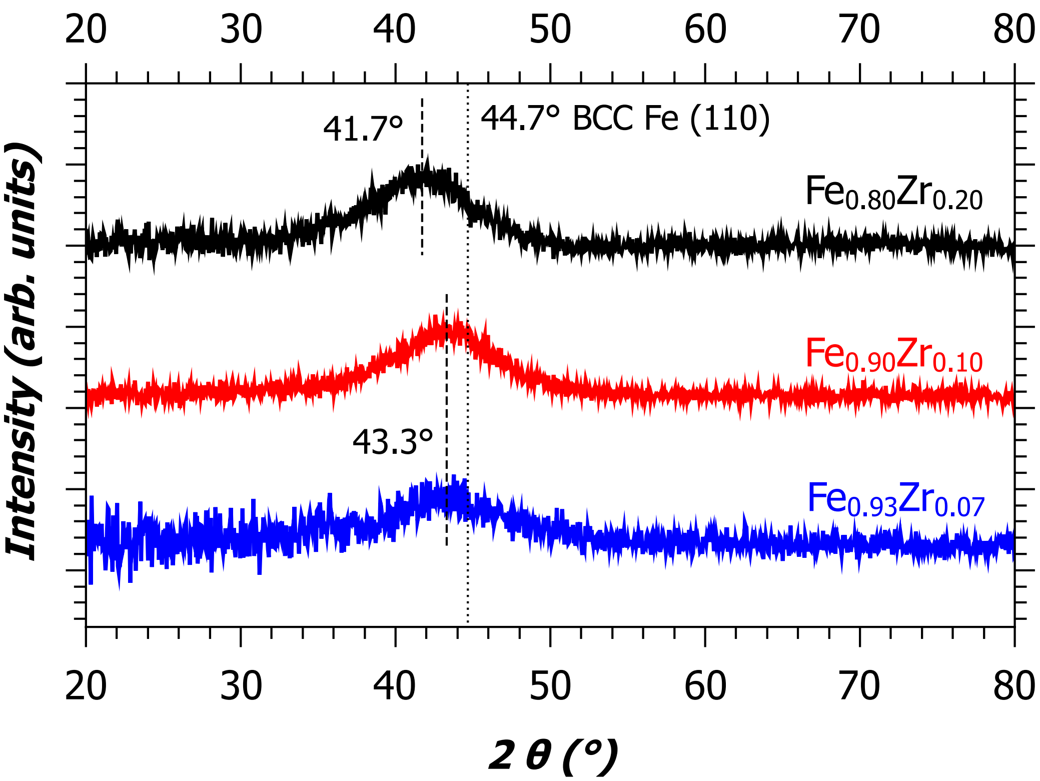

FIG. 1. Grazing incidence x-ray diffraction (GIXRD) intensity versus 2θ after background subtraction. The (110) peak position of BCC iron is included for reference.

TABLE I. The number of Fe and Zr atoms in each supercell consisting of 200 atoms in total. The sizes of the supercells are calculated from the number density, ρ_N, values reported in the literature [1, 2].

Composition	Fe atoms	Zr atoms	ρ_N (Å^{-3})	Supercell size (Å)
$Fe_{0.93}Zr_{0.07}$	186	14	0.080	13.55
$Fe_{0.90}Zr_{0.10}$	180	20	0.078	13.66
$Fe_{0.80}Zr_{0.20}$	160	40	0.073	14.01

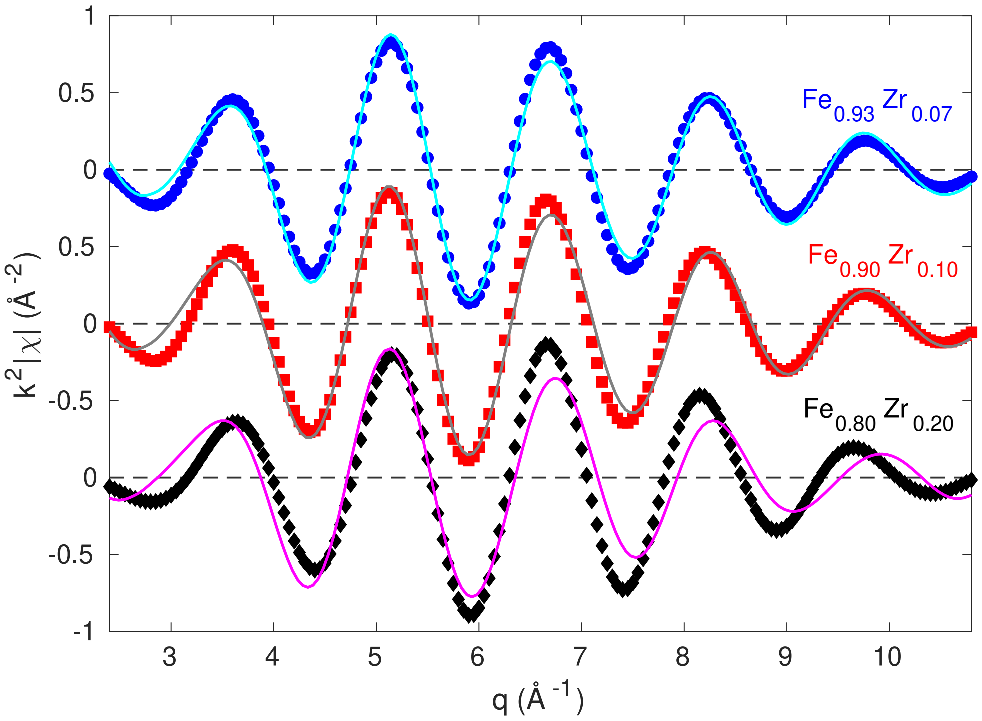

FIG. 2. k^2-weighted real part of χ for experimental data (points) measured at 80 K and simulated data (lines). Both the experimental and simulated data are limited to the first shell. The back-Fourier analysis is made for $R \sim 1.5 - 2.7$ Å using a Hanning window for the experimental data (corresponding to non-phase corrected distances). The simulated EXAFS signal is calculated by averaging the Fe K-edge $\chi_i(k)$ signal from all $200(1-x)$ Fe atoms in each of the $Fe_{(1-x)}Zr_x$ supercells. A cut-off is made in the calculation of $\chi(k)$ at $R_{\max} = 3.2$ Å corresponding to the minimum in the total RDF. The amplitude factor $S_0^2 = 0.67$, 0.73, and 0.87 and the energy shift $\Delta E_0 = 4.2$, 2.8, and 0.3 for $Fe_{0.93}Zr_{0.07}$, $Fe_{0.90}Zr_{0.10}$, and $Fe_{0.80}Zr_{0.20}$, respectively. The data are shifted for clarity with the dashed lines indicating each zero level.

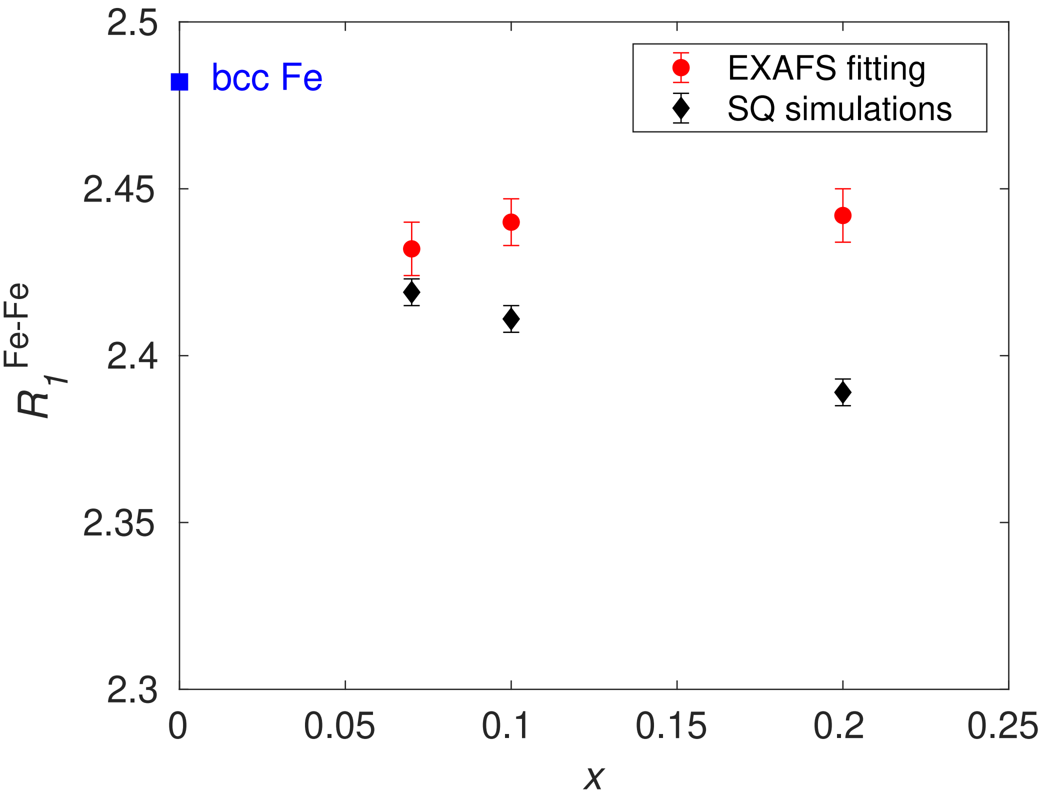

FIG. 3. The $R_1^{\mathrm{Fe-Fe}}$ distances obtained in by EXAFS fitting (circles) and SQ-simulations (diamonds) as a function of Zr content, x, in $Fe_{(1-x)}Zr_x$.

TABLE II. The nearest neighbor Fe-Fe distance, $R_1^{\mathrm{Fe-Fe}}$, obtained in the EXAFS analysis presented in Table 4 and Curie temperature, T_c. For the $Fe_{(1-x)}Zr_x$ samples, T_c is measured in a longitudinal magneto-optical Kerr effect (L-MOKE) setup [3], while the T_c value for BCC Fe is taken from the literature [4].

Composition	$R_1^{\mathrm{Fe-Fe}}$ (Å)	T_c (K)
$Fe_{0.93}Zr_{0.07}$	2.432(8)	144(1)
$Fe_{0.90}Zr_{0.10}$	2.440(7)	265(1)
$Fe_{0.80}Zr_{0.20}$	2.442(8)	280(1)
Fe (BCC)	2.481(5)	1044 [4]

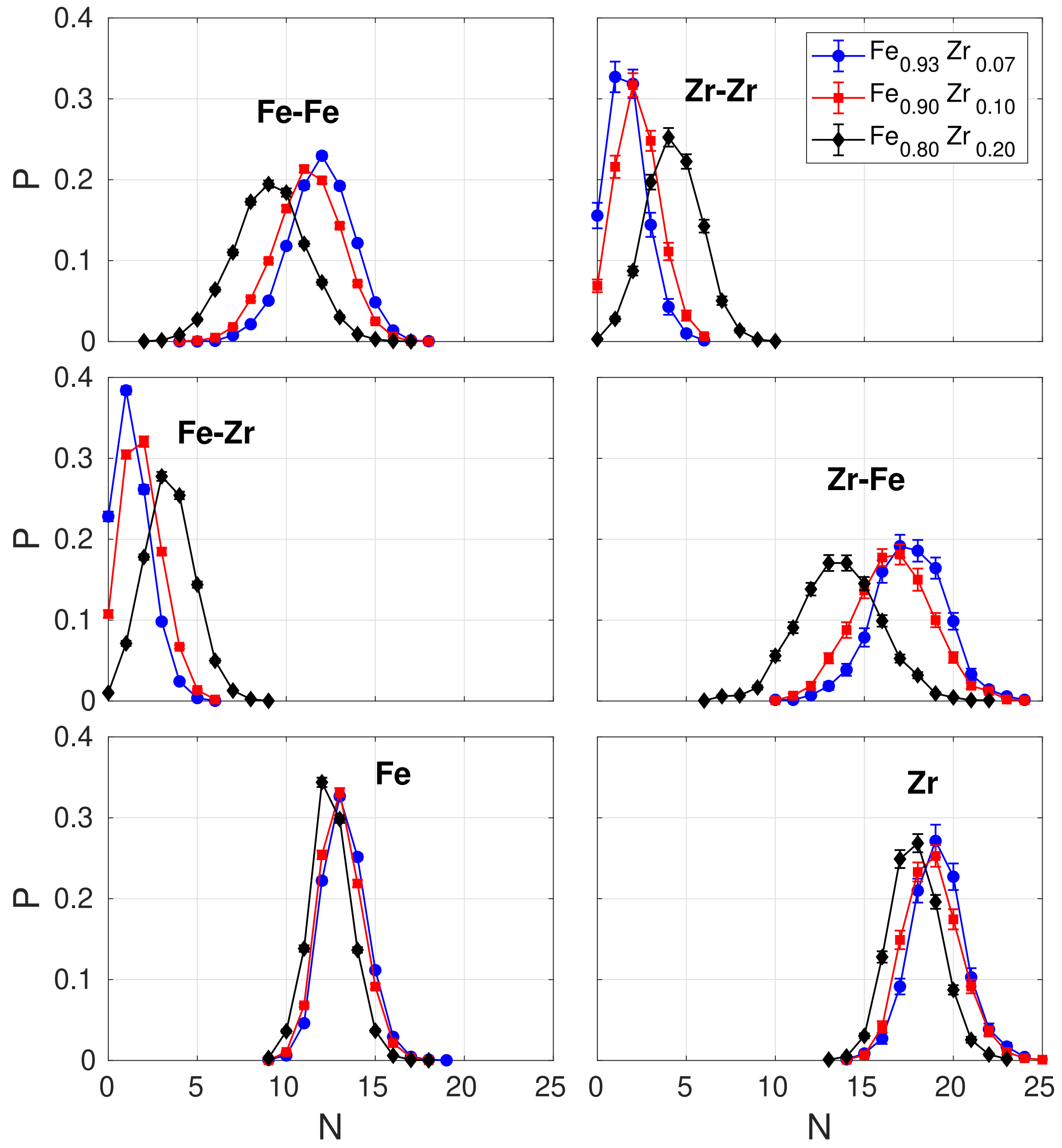

FIG. 4. Fraction of partial coordination numbers obtained by Voronoi analysis using the Voro++ software package [5]. The difference in size between the Fe and Zr atoms is taken into account when creating the Voronoi polyhedra. Voronoi polyhedra surfaces with an area that comprises less than 1% of the total area of the polyhedra are not included.

[1] S. Krompiewski, U. Krauss, and U. Krey, "Computer studies of magnetization for amorphous $Fe_{1-x}Zr_xH_y$ alloys," *Phys. Rev. B*, vol. 39, pp. 2819–2821, 1989. doi:10.1103/PhysRevB.39.2819.

[2] I. Bakonyi, "Atomic volumes and local structure of metallic glasses," *Acta Mater.*, vol. 53, pp. 2509–2520, 2005. doi:10.1016/j.actamat.2005.02.016.

[3] G. Muscas, R. Brucas, and P. E. Jönsson, "Bringing nanomagnetism to the mesoscale with artificial amorphous structures," *Phys. Rev. B*, vol. 97, pp. 1–8, 2018. doi:10.1103/PhysRevB.97.174409.

[4] J. M. D. Coey, *Magnetism and Magnetic Materials.* Cambridge University Press, 2010. doi:10.1017/CBO9780511845000.

[5] C. H. Rycroft, "VORO++ : A three-dimensional Voronoi cell library in C++," *Chaos An Interdiscip. J. Nonlinear Sci.*, vol. 19, p. 041111, 2009. doi:10.1063/1.3215722.
